# HOW I DO IT: Breaking boundaries in surgical education by delivering expert feedback to residents anytime and anywhere. The LAPPCLINIC project.

**DOI:** 10.1016/j.sopen.2025.09.007

**Published:** 2025-09-12

**Authors:** Diego Sanhueza R., Cristián Jarry T., Julián Varas C.

**Affiliations:** a Experimental Surgery and Simulation Center, Faculty of Medicine, Pontificia Universidad Católica de Chile, Santiago, Chile; b Colorectal Surgery Unit, Department of Digestive Surgery, Pontificia Universidad Catolica de Chile, Santiago, Chile; c Department of Digestive Surgery, Pontificia Universidad Católica de Chile, Santiago, Chile

**Keywords:** Video-based assessment, Surgical education, Feedback, Remote feedback

## Abstract

**Objective:**

To describe *LAPPCLINIC*, an innovative web-based platform designed to enhance surgical education through remote and asynchronous feedback by video-analysis of residents' own surgical procedures.

**Design:**

We provide a detailed description of the platform workflow, highlighting key features for enhancing surgical education.

**Setting:**

An ongoing multicenter study involving seven surgical residency programs across Chile.

**Participants:**

First-year surgical residents from seven different Chilean programs, with feedback provided by five surgeons, experienced in surgical education, who are beyond their learning curves in laparoscopic cholecystectomy and trained in structured quality-feedback delivery.

**Conclusion:**

*LAPPCLINIC* implementation has shown strong resident acceptance and significantly higher evaluation of feedback quality compared to traditional OR-based teaching.

## Highlights

• Video Based Assessment (VBA) for enhancing surgical education
• Remote, asynchronous expert feedback of residents recorded surgeries
• Platform-based feedback enables scalable surgical education through VBA.
• Residents rated deferred feedback as higher quality than feedback given in the OR.

## Introduction

Surgical education has continuously evolved, adapting to the changing needs of residents and healthcare systems [[1], [2], [3]]. Traditional mentor-apprentice approach, face challenges due to reduced operative exposure, resulting of a growing number of residents, the increasing demand of subspecialists formation, work-hour restrictions and an increased focus on patient safety [3,4]. Therefore, educational strategies are shifting from a teacher-centered to a learner-centered approach, integrating innovative methods to complement and enhance operating room (OR) experiences [[4], [5], [6]].

Effective feedback is essential for skill development, particularly when it is specific, timely, and provided in a supportive environment [2]. Learning opportunities in the OR are inherently stressful: residents are highly aware of the risk of making mistakes, and time pressures often limit comprehensive feedback [2,4,7]. Additionally, faculty feedback in the OR is not always perceived as such by residents, limiting intraoperative learning [8,9]. This scenario provides an opportunity to complement OR-based teaching with remote, asynchronous feedback, allowing residents to review and reflect on their performances at their own pace, reinforcing key learning points outside the stress of real-time surgery [[10], [11], [12], [13]].

Asynchronous feedback models have shown promising results in simulation-based training, and studies now apply similar methods for video review of actual surgical cases [6,10,[14], [15], [16], [17]]. Moreover, digital media and e-learning have proven especially effective for surgical education, offering flexible and accessible solutions that allow continuous practice, feedback, and skill improvement [18,19].

In this context, *LAPPCLINIC* was developed, a novel web-based platform designed to improve residents' surgical performance through video analysis of their own real-case procedures. Integrating remote and asynchronous expert feedback to real life surgical videos performed by residents. Allowing them to effectively identify and correct mistakes, ultimately optimizing the quality of their operative training [2,15].

This article outlines the conception, implementation, and future directions of *LAPPCLINIC* as an innovative educational tool in surgical training.

## Methods

### Platform design and architecture

**Fig. 1:**
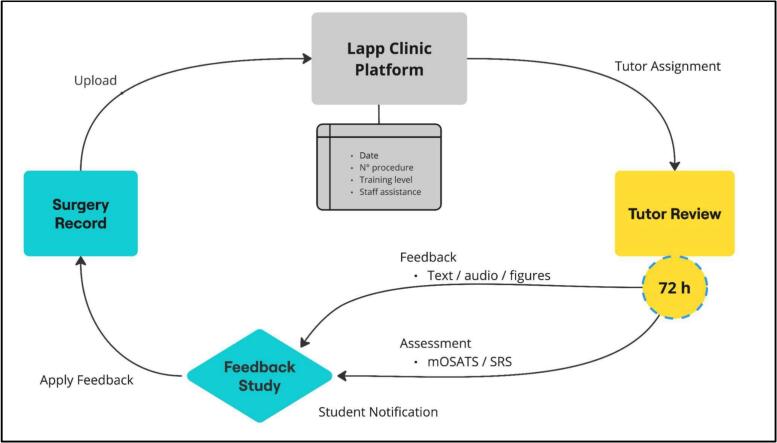
Workflow of LAPPCLINIC platform.

*LAPPCLINIC* workflow begins with residents recording surgical procedures and then uploading their videos to the platform (Fig. 1), only surgical data is collected, and no patient data is requested. Upon uploading a video, tutors receive an automatic email notification prompting them to assess resident's performance using structured and validated evaluation tools. Then videos are analysed, assessed and asynchronous feedback is delivered by assigned tutors. After video assessment, resident receives a notification to review his performance and feedback alongside the video. The platform interface synchronizes the feedback provided with specific frames of residents' video, reinforcing learning with context.

### Innovative features and personalized feedback

*LAPPCLINIC* incorporates advanced educational tools to enhance feedback quality, such as checklists, Likert scales, and pinpoint timestamps in any specific video frame to highlight technical errors. Feedback is delivered through multimodal formats, including written annotations, voice memo-recordings, and drawings or graphical annotations directly on the video, allowing for precise and step-specific suggestions for improvement.

### Patient privacy

*LAPPCLINIC* fully complies with local regulations and is designed to meet HIPAA requirements. Before uploading surgical videos to the platform, our informatic team or residents pre-process them by trimming the beginning and end to remove any sensitive patient information or unnecessary footage, and by compressing videos for efficient uploading.

### How we do it: a national multicenter study

#### Participants

Trainees: Residents of seven different surgical training programs across Chile. They were recruited at the beginning of their surgical program since 2022 and followed up.

Tutors: Experienced surgeons with background in surgical education, trained in feedback delivery in laparoscopic cholecystectomy.

- Surgeons who have completed their learning curve for laparoscopic cholecystectomy, having performed more than 300, and currently performing over 50 per year.
- To ensure consistency and objectivity among assessments, all tutors underwent a calibration process before participating in the study. It included training on validated assessment tools (mOSATS and OPRS), independent video evaluations, and iterative consensus meetings, ensuring an inter-rater reliability >0.8 and reproducible assessments.

#### The LAPPCLINIC project

Trainees record their laparoscopic cholecystectomy surgeries using standard OR equipment or portable recording devices. Then, they upload their videos to *LAPPCLINIC* and provide surgical data, such as the number of cholecystectomies previously performed, prior simulation training, level of staff assistance, and case difficulty.

Instructors receive an email notification when a trainee uploads a video and are expected to provide feedback within 72 h. Resident performance is assessed using a modified *Objective Structured Assessment of Technical Skill* score (mOSATS) and the *Operative Performance Rating System* (OPRS) of the *American Board of Surgery* [20].

**Fig. 2:**
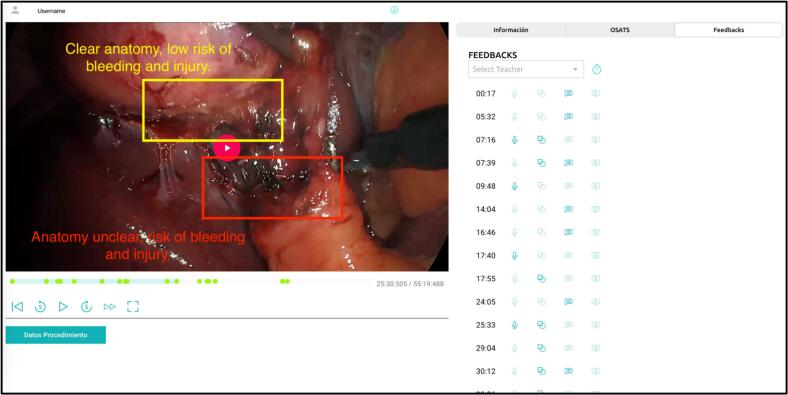
Tutor interface in LAPPCLINIC platform evaluating a resident's laparoscopic cholecystectomy. Green dots in the video-timeline indicate where feedback was provided, with details accessible on the right side. (For interpretation of the references to colour in this figure legend, the reader is referred to the web version of this article.)

Once the assessment is completed, residents receive an email notification with a direct link to access their performance review (Fig. 2). They can revisit their video assessment at any time, as all evaluations remain stored in their website profile. Additionally, *LAPPCLINIC* features a dashboard that tracks residents' progress, displaying performance trends and the number of uploaded surgeries, enabling both trainees and educators to monitor skill development over time.

#### Survey analysis

We sent an online survey to all 86 residents participating in the study. The survey collected residents' opinions through Likert-scale questions covering demographic data, feedback assessment, barriers to project participation, platform usability, and perceived impact. Data were coded and analysed using descriptive statistics.

## Results

Preliminary results of user perception indicates a high acceptance of *LAPPCLINIC* among surgical residents, 28 residents (33 %) answered the survey with 91 % recommending its use and 81 % reporting self-improvements in surgical skills. Feedback received through the platform was rated significantly higher than traditional intraoperative feedback (6.05 vs. 5.25 out of 7, *p* = 0.0065), highlighting its perceived educational value. Trainees reported that the platform was intuitive and easy to navigate. Primary challenges identified included memory card problems (54 %), time constraints (39 %) and recording devices problems (35 %).

### Limitations

Despite positive findings, our study has several limitations to discuss. First, laparoscopic cholecystectomy techniques vary across institutions; depending on different surgical “schools”, center resource availability and individual staff preferences; leading to heterogeneous resident performance that may confound residents performance. Second, asynchronous review makes it difficult to determine whether intraoperative decisions were made independently by the resident or guided by the staff surgeon. Third, we observed significant delays between case recording and video upload which could affect both study outcomes and the accuracy of surgical data labeling. Finally, in this pilot we analysed every uploaded video, but as LAPPCLINIC scales to more trainees and procedures, it will be important to assess whether sampling a subset of cases can deliver educational benefits without overloading tutors. We plan to address some of these issues in a future study with an in-depth analysis of resident performance and platform usability data.

Currently we are exploring the impact of remote and asynchronous feedback in surgical learning curve, by assessing whether residents who receive feedback through *LAPPCLINIC* achieve higher competency levels compared to those trained exclusively through traditional OR-based learning with in-person supervision.

## Conclusions

*LAPPCLINIC* leverages individualized, remote and asynchronous feedback to address the growing demands of modern surgical education. Early findings suggest high user acceptance, self-perceived skill improvement, and favorable comparisons to traditional OR-based feedback. By complementing traditional OR-based feedback, *LAPPCLINIC* may help standardize competencies across diverse training programs. It overcomes geographical barriers by connecting trainees with expert instructors regardless of location and mitigates tutor-dependent constraints by ensuring standardized, high-quality feedback.

### Scalability & future directions

Asynchronous feedback allows a single mentor to oversee multiple trainees efficiently, making it particularly valuable in resource-limited settings and high-demand training programs [13,14,16]. This approach aligns with recommendations from international surgical education panels, which emphasize that well-implemented technology can alleviate faculty workload while maintaining training quality [15,[21], [22], [23]].

We seek to expand *LAPPCLINIC* by incorporating a broader range of surgical procedures and broadening the platform's scope as a comprehensive training ecosystem for different specialities. To boost resident engagement and highlight the platform's value, we are working on automating the video-upload process.

Artificial intelligence (AI) integration on digital platforms presents a significant opportunity to enhance surgical education. Machine learning models have demonstrated the ability to evaluate technical proficiency by recognizing motion patterns, error detection and performance trends, potentially offering an additional layer of guidance beyond expert review [24,25]. AI-driven analytics could support automated skill assessment, eventually providing real-time intraoperative assessment, functioning as virtual coaches to guide trainees and surgeons through surgical tasks [[26], [27], [28]].

As surgical training continues to evolve, embracing innovation, digital mentorship and learner-centered strategies like *LAPPCLINIC* will be critical in optimizing learning outcomes and broadening access to expert guidance [1,4,15,21,29].

## CRediT authorship contribution statement

**Diego Sanhueza R.:** Writing – original draft, Project administration, Investigation, Formal analysis. **Cristián Jarry T.:** Writing – original draft, Project administration, Methodology, Investigation, Formal analysis. **Julián Varas C.:** Supervision, Project administration, Methodology, Investigation, Funding acquisition, Conceptualization.

## Ethics approval

This study was approved by the Pontificia Universidad Católica de Chile Faculty of Medicine Ethical Committee and by the ethics committees of all participating training centers.

## Funding sources

Funding was obtained through Chile's National Agency for Research and Development, 10.13039/501100002850FONDECYT Regular 2022, No. 1221490, ANID.

## Declaration of competing interest

Dr. Julián Varas is the Founder and Chief Executive Officer of Training Competence, an official spin-off of the Pontificia Universidad Católica de Chile behind the LAPPCLINIC platform. Created for educational and research use, the platform was offered free of charge to all participants in the aforementioned study. Dr. Sanhueza and Dr. Jarry have no conflicts of interest to disclose.
